# Aqua­(2,9-dimethyl-1,10-phenanthroline-κ^2^ *N*,*N*′)diformato-κ^2^ *O*,*O*′;κ*O*-nickel(II) monohydrate

**DOI:** 10.1107/S1600536811030558

**Published:** 2011-08-02

**Authors:** Ping Xia, Jian-Li Lin, Sheng-Liang Ni

**Affiliations:** a Department of Chemistry, Huzhou Teachers College, Huzhou, Zhejiang 313000, People’s Republic of China; b Center of Applied Solid State Chemistry Research, Ningbo University, Ningbo 315211, People’s Republic of China

## Abstract

The asymmetric unit of the title compound, [Ni(HCO_2_)_2_(C_14_H_12_N_2_)(H_2_O)]·H_2_O, contains a mononuclear complex mol­ecule hydrogen bonded to a lattice water mol­ecule. The Ni^II^ atom exhibits a distorted octa­hedral coordination geometry formed by the N atoms from a 2,9-dimethyl-1,10-phenanthroline ligand, two O atoms of a chelating formate anion, one aqua O atom and one O atom of a coordinating formate anion. The mol­ecules are assembled into chains extending along [100] through by O—H⋯O hydrogen bonds. The supra­molecular chains are further linked into layers parallel to (011) by weak π–π packing inter­actions [centroid–centroid separation = 3.768 (2) Å]. The resulting layers are stacked to meet the requirement of close-packing patterns.

## Related literature

For general background to supra­molecular architectures, see: Moulton & Zaworotko (2001 ▶); Aakeroy & Seddon (1993 ▶). For related structures, see: Go *et al.* (2004 ▶); Wang *et al.* (2006) ▶; Ni *et al.* (2011 ▶).
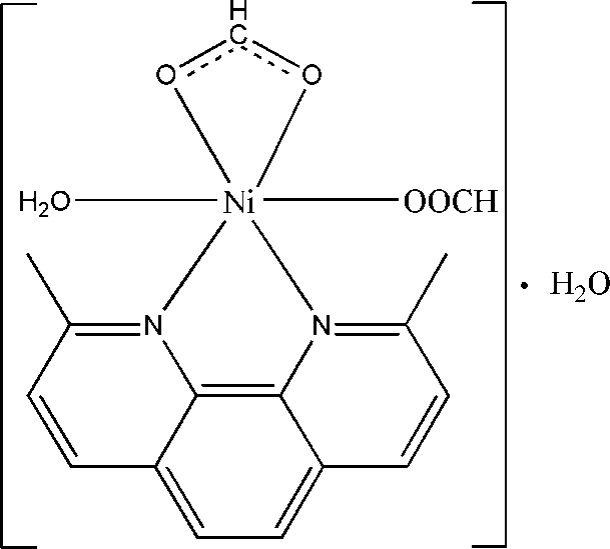


## Experimental

### 

#### Crystal data

[Ni(HCO_2_)_2_(C_14_H_12_N_2_)(H_2_O)]·H_2_O
*M* *_r_* = 393.03
Triclinic, 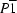

*a* = 7.3992 (15) Å
*b* = 10.373 (2) Å
*c* = 11.442 (2) Å
α = 82.42 (3)°
β = 81.77 (3)°
γ = 76.10 (3)°
*V* = 839.3 (3) Å^3^
*Z* = 2
Mo *K*α radiation
μ = 1.19 mm^−1^
*T* = 298 K
0.30 × 0.20 × 0.15 mm

#### Data collection

Rigaku R-AXIS RAPID diffractometer
Absorption correction: multi-scan (*ABSCOR*; Higashi, 1995 ▶) *T*_min_ = 0.750, *T*_max_ = 0.821
8265 measured reflections
3785 independent reflections
3214 reflections with *I* > 2σ(*I*)
*R*_int_ = 0.025

#### Refinement

*R*[*F* ^2^ > 2σ(*F* ^2^)] = 0.035
*wR*(*F* ^2^) = 0.118
*S* = 1.22
3785 reflections
226 parameters
H-atom parameters constrained
Δρ_max_ = 0.69 e Å^−3^
Δρ_min_ = −0.67 e Å^−3^

### 

Data collection: *RAPID-AUTO* (Rigaku, 1998 ▶); cell refinement: *RAPID-AUTO*; data reduction: *CrystalStructure* (Rigaku/MSC, 2004 ▶); program(s) used to solve structure: *SHELXS97* (Sheldrick, 2008 ▶); program(s) used to refine structure: *SHELXL97* (Sheldrick, 2008 ▶); molecular graphics: *SHELXTL* (Sheldrick, 2008 ▶); software used to prepare material for publication: *SHELXTL*.

## Supplementary Material

Crystal structure: contains datablock(s) global, I. DOI: 10.1107/S1600536811030558/zk2016sup1.cif

Structure factors: contains datablock(s) I. DOI: 10.1107/S1600536811030558/zk2016Isup2.hkl

Additional supplementary materials:  crystallographic information; 3D view; checkCIF report

## Figures and Tables

**Table 1:** Hydrogen-bond geometry (Å, °)

*D*—H⋯*A*	*D*—H	H⋯*A*	*D*⋯*A*	*D*—H⋯*A*
O5—H51⋯O4^i^	0.86	1.85	2.703 (3)	173.3
O5—H52⋯O6^ii^	0.84	2.03	2.808 (4)	154.2
O6—H61⋯O2^iii^	0.85	1.98	2.830 (4)	179.3
O6—H62⋯O3^iv^	0.85	2.43	3.077 (4)	133.4

Symmetry codes: (i) 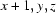; (ii) 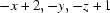; (iii) 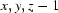; (iv) 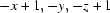.

**Fig. 1.**
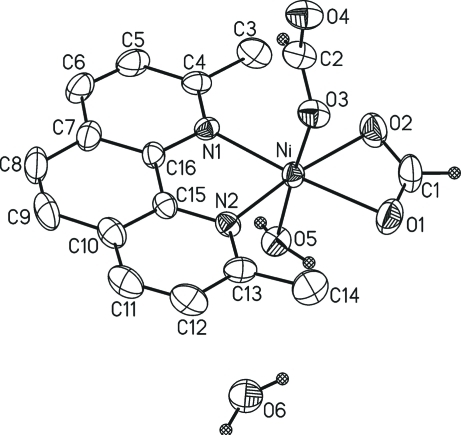
ORTEP view of the title compound. The dispalcement ellipsoids are drawn at 45% probability level.

**Fig. 2.**
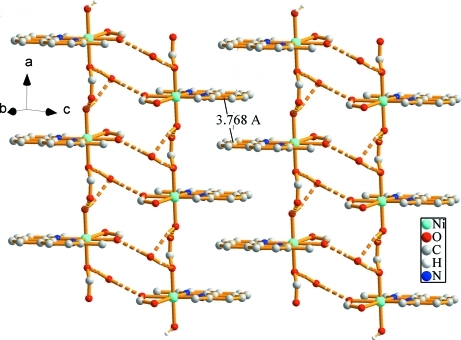
two-dimensional layer structure link through hydrogen bonds and π–π packing interactions of the title compound

## supplementary crystallographic information

### Comment

In the past decade, a variety of supramolecular architectures based on non–covalent intermolecular interactions such as hydrogen bonding, van der Walls forces and π–π interactions have been achieved by using transition metal centers and organic ligands due to their possible intriguing structural topologies and potential applications in optics, catalysis, ion exchange, gas storage, and the molecular–based magnetic materials (Aakeroy & Seddon, 1993). Carboxylate ligands have been actively utilized as construction units to obtain lots of supramolecular complexes (Moulton & Zaworotko, 2001). In the paper, we are interested in self–assemblies of Ni^II^ ions and 2,9'–dimethyl–1,10'–phenanthroline with formic acid, leading to successful preparation of a complex [Ni(HCO_2_)_2_(C_14_H_12_N_2_)(H_2_O)].H_2_O. The asymmetric unit of the title compound consists of one Ni^II^ ion, one H_2_O molecule, one 2,9'–dimethyl–1,10'–phenanthroline molecule, one O,*O*'–chelated formate anion and another coordinated formate anion, and one lattice H_2_O molecule(Fig. 1). It's worth to mention, the tetragonal plane is built up by a pair of bidentate formate anions using carboxyl oxygen atoms (Ni–O1 = 2.148 (3) Å, Ni–O2 = 2.150 (3) Å) and by a neutral 2,9'–dimethyl–1,10'–phenanthroline molecule using nitrogen atoms(Ni–N1 = 2.087 (3) Å, Ni–N2 = 2.076 (3) Å).(Table.1). The four atoms around Ni^II^ are almost coplanar and show deviations from -0.093 (2) to 0.092 (2) Å with the average plane, the axial positions are occupied by a pair of oxygen originating from H_2_O (Ni–O5 = 2.067 (3) Å) and formate anion (Ni–O3 = 2.046 (2) Å), significantly shorter than the Ni–O1 or Ni–O2 bond distance, which are similar to those observed in related complexes with carboxylate complexes(Go *et al.*, 2004; Wang *et al.*, 2006; Ni *et al.*, 2011). The selected bond angles of O1–Ni–O2, N1–Ni–N2, O3–Ni–O5 are 81.5 (1) °, 61.0 (1) °, and 175.9 (1) °, respectively. For two formate anions, the angle (O1–C1–O2, 122.0 (3) °) of chelated formate is smaller than coordinated (O3–C2–O4, 127.6 (3) Å). The 2,9'–dimethyl–1, 10'–phenanthroline ligand are almost coplanar with the mean square deviations 0.0158 Å, the water molecule is not coordinated to Ni atom and the distance between nickel and water oxygen atom of 7.146 (2) Å.

The molecules are assembled into one-dimensional chains extending along the [100] direction through O5–H51···O4^#1^, O5–H52···O6^#2^, O6–H61···O2^#3^ and O6–H61···O3^#4^ hydrogen bonds(#1 = *x* + 1, *y*, *z*; #2 = -*x* + 2, -*y*, -*z* + 1; #3 = *x*, *y*, *z* - 1; #4 = -*x* + 1, -*y*, -*z* + 1)(Table.2), the double chains are further linked into two-dimensional layers which parallel to (011) by weak π–π packing interaction(ring centroid separation, 3.768 (2) Å).(Fig. 2). The resulting layers are stacked to meet the requirement of close–packing patterns.

### Experimental

Dropwise addition of 2.0 ml of 1.0 mol.l^-1^ aqueous Na_2_CO_3_ to a stirred aqueous solation of NiSO_4_.7H_2_O (0.2876 g, 1.0 mmol) in 5.0 ml H_2_O produced a green precipitate, Ni(OH)_2–2x_(CO_3_)*_x_*.yH_2_O, which was centrifuged and washed with water until no SO_4_^2-^ anions were detected in the supernatant. The precipitate was added to a stirred aqueous ethanolic solution of 2,9'–dimethyl–1,10'–phenanthroline in 15 ml EtOH–H_2_O (2:1,*v*/*v*). And then, 2.0 ml of 1.0 mol.l^-1^aqueous formalic acid was dropwise added to above mixture and stirred continuously until dissolved of the green precipitate. The green solution (pH = 3.37) was allowed to stand at room temperature. Slow evaporation during two weeks afforded green block crystals(yield:42%).

### Refinement

All H–atoms bonded to C were positioned geometrically and refined using a riding model with d(C–H) = 0.093 Å calculated positionand were refined using a riding, *U*_iso_(H) = 1.2 *U*_eq_(C) for aromatic, 0.93 Å, *U*_iso_(H) = 1.2 *U*_eq_(C) for CH and 0.96 Å, *U*_iso_(H) = 1.5 *U*_eq_(C) for CH_3_ atoms. H atoms attached to O atoms were found in a difference Fourier synthesisand were refined using a riding model, with the O–H distances fixed as initially found and with *U*_iso_(H) values set at 1.2 *U*eq(O).

### Crystal data

**Table:**

[Ni(HCO_2_)_2_(C_14_H_12_N_2_)(H_2_O)]·H_2_O	*Z* = 2
*M**_r_* = 393.03	*F*(000) = 408
Triclinic, *P*1	*D*_x_ = 1.555 Mg m^−^^3^
Hall symbol: -P 1	Mo *K*α radiation, λ = 0.71073 Å
*a* = 7.3992 (15) Å	Cell parameters from 25 reflections
*b* = 10.373 (2) Å	θ = 3.1–27.4°
*c* = 11.442 (2) Å	µ = 1.19 mm^−^^1^
α = 82.42 (3)°	*T* = 298 K
β = 81.77 (3)°	Block, green
γ = 76.10 (3)°	0.30 × 0.20 × 0.15 mm
*V* = 839.3 (3) Å^3^	

### Data collection

**Table:**

Rigaku R-AXIS RAPID diffractometer	3785 independent reflections
Radiation source: fine-focus sealed tube	3214 reflections with *I* > 2σ(*I*)
graphite	*R*_int_ = 0.025
ω scans	θ_max_ = 27.4°, θ_min_ = 3.1°
Absorption correction: multi-scan (*ABSCOR*; Higashi, 1995)	*h* = −9→8
*T*_min_ = 0.750, *T*_max_ = 0.821	*k* = −13→13
8265 measured reflections	*l* = −14→14

### Refinement

**Table:**

Refinement on *F*^2^	Secondary atom site location: difference Fourier map
Least-squares matrix: full	Hydrogen site location: inferred from neighbouring sites
*R*[*F*^2^ > 2σ(*F*^2^)] = 0.035	H-atom parameters constrained
*wR*(*F*^2^) = 0.118	*w* = 1/[σ^2^(*F*_o_^2^) + (0.0328*P*)^2^ + 1.2744*P*] where *P* = (*F*_o_^2^ + 2*F*_c_^2^)/3
*S* = 1.22	(Δ/σ)_max_ = 0.001
3785 reflections	Δρ_max_ = 0.69 e Å^−^^3^
226 parameters	Δρ_min_ = −0.67 e Å^−^^3^
0 restraints	Extinction correction: *SHELXL*
Primary atom site location: structure-invariant direct methods	Extinction coefficient: 0.0015 (3)

### Special details

**Table:**

Geometry. All e.s.d.'s (except the e.s.d. in the dihedral angle between two l.s. planes) are estimated using the full covariance matrix. The cell e.s.d.'s are taken into account individually in the estimation of e.s.d.'s in distances, angles and torsion angles; correlations between e.s.d.'s in cell parameters are only used when they are defined by crystal symmetry. An approximate (isotropic) treatment of cell e.s.d.'s is used for estimating e.s.d.'s involving l.s. planes.
Refinement. Refinement of *F*^2^ against ALL reflections. The weighted *R*-factor *wR* and goodness of fit *S* are based on *F*^2^, conventional *R*-factors *R* are based on *F*, with *F* set to zero for negative *F*^2^. The threshold expression of *F*^2^ > σ(*F*^2^) is used only for calculating *R*-factors(gt) *etc*. and is not relevant to the choice of reflections for refinement. *R*-factors based on *F*^2^ are statistically about twice as large as those based on *F*, and *R*- factors based on ALL data will be even larger.

### Fractional atomic coordinates and isotropic or equivalent isotropic displacement parameters (Å^2^)

**Table:**

	*x*	*y*	*z*	*U*_iso_*/*U*_eq_	
Ni	0.77473 (5)	0.18268 (4)	0.77154 (3)	0.02776 (13)	
O1	0.8178 (4)	−0.0310 (2)	0.7920 (3)	0.0540 (7)	
O2	0.7827 (4)	0.0786 (3)	0.9468 (2)	0.0494 (6)	
C1	0.8093 (6)	−0.0283 (4)	0.9011 (4)	0.0535 (10)	
H1A	0.8232	−0.1082	0.9500	0.064*	
O3	0.4915 (3)	0.1977 (2)	0.7917 (2)	0.0395 (5)	
O4	0.2105 (3)	0.3030 (3)	0.8671 (2)	0.0485 (6)	
C2	0.3833 (5)	0.2772 (4)	0.8554 (3)	0.0418 (8)	
H2A	0.4386	0.3228	0.8997	0.050*	
O5	1.0628 (3)	0.1559 (2)	0.7442 (2)	0.0378 (5)	
H52	1.1256	0.0773	0.7581	0.045*	
H51	1.1024	0.2025	0.7876	0.045*	
O6	0.6484 (4)	0.0613 (3)	0.1914 (2)	0.0544 (7)	
H61	0.6881	0.0662	0.1176	0.065*	
H62	0.5887	0.0096	0.2356	0.065*	
N1	0.7660 (3)	0.3828 (2)	0.7853 (2)	0.0271 (5)	
N2	0.7565 (4)	0.2549 (3)	0.5945 (2)	0.0296 (5)	
C3	0.7791 (6)	0.3669 (4)	0.9991 (3)	0.0460 (9)	
H3A	0.7739	0.2766	0.9924	0.069*	
H3B	0.6727	0.4074	1.0509	0.069*	
H3C	0.8922	0.3672	1.0310	0.069*	
C4	0.7770 (4)	0.4441 (3)	0.8789 (3)	0.0334 (7)	
C5	0.7852 (5)	0.5793 (4)	0.8660 (4)	0.0456 (9)	
H5A	0.7951	0.6194	0.9321	0.055*	
C6	0.7789 (6)	0.6518 (4)	0.7588 (4)	0.0493 (9)	
H6A	0.7825	0.7415	0.7516	0.059*	
C7	0.7668 (5)	0.5908 (3)	0.6584 (3)	0.0393 (7)	
C8	0.7604 (6)	0.6596 (4)	0.5418 (4)	0.0530 (10)	
H8A	0.7629	0.7496	0.5305	0.064*	
C9	0.7509 (6)	0.5968 (4)	0.4485 (4)	0.0527 (10)	
H9A	0.7458	0.6441	0.3736	0.063*	
C10	0.7485 (5)	0.4584 (4)	0.4624 (3)	0.0412 (8)	
C11	0.7418 (6)	0.3871 (5)	0.3671 (3)	0.0530 (10)	
H11A	0.7382	0.4300	0.2906	0.064*	
C12	0.7406 (6)	0.2557 (5)	0.3877 (3)	0.0530 (10)	
H12A	0.7360	0.2085	0.3249	0.064*	
C13	0.7465 (5)	0.1901 (4)	0.5032 (3)	0.0392 (7)	
C14	0.7372 (7)	0.0465 (4)	0.5273 (4)	0.0602 (11)	
H14A	0.7439	0.0183	0.6102	0.090*	
H14B	0.8404	−0.0066	0.4815	0.090*	
H14C	0.6214	0.0358	0.5057	0.090*	
C15	0.7550 (4)	0.3872 (3)	0.5750 (3)	0.0306 (6)	
C16	0.7622 (4)	0.4551 (3)	0.6761 (3)	0.0301 (6)	

### Atomic displacement parameters (Å^2^)

**Table:**

	*U*^11^	*U*^22^	*U*^33^	*U*^12^	*U*^13^	*U*^23^
Ni	0.0320 (2)	0.0254 (2)	0.0280 (2)	−0.01022 (15)	−0.00438 (15)	−0.00207 (14)
O1	0.078 (2)	0.0316 (13)	0.0548 (17)	−0.0161 (13)	−0.0093 (14)	−0.0044 (11)
O2	0.0675 (18)	0.0447 (15)	0.0364 (13)	−0.0178 (13)	−0.0080 (12)	0.0058 (11)
C1	0.070 (3)	0.0368 (19)	0.052 (2)	−0.0164 (18)	−0.0105 (19)	0.0139 (17)
O3	0.0273 (11)	0.0512 (14)	0.0448 (13)	−0.0162 (10)	−0.0022 (10)	−0.0107 (11)
O4	0.0317 (13)	0.0651 (17)	0.0524 (15)	−0.0129 (12)	−0.0027 (11)	−0.0177 (13)
C2	0.0361 (18)	0.056 (2)	0.0394 (18)	−0.0179 (16)	−0.0018 (14)	−0.0144 (16)
O5	0.0293 (11)	0.0398 (12)	0.0462 (13)	−0.0070 (9)	−0.0104 (10)	−0.0054 (10)
O6	0.0642 (18)	0.0550 (16)	0.0460 (15)	−0.0148 (14)	−0.0076 (13)	−0.0080 (12)
N1	0.0247 (12)	0.0272 (12)	0.0303 (12)	−0.0059 (10)	−0.0023 (9)	−0.0072 (9)
N2	0.0316 (13)	0.0338 (13)	0.0248 (12)	−0.0104 (11)	−0.0033 (10)	−0.0028 (10)
C3	0.052 (2)	0.061 (2)	0.0322 (17)	−0.0201 (18)	−0.0079 (15)	−0.0130 (16)
C4	0.0290 (15)	0.0397 (17)	0.0350 (16)	−0.0114 (13)	0.0011 (12)	−0.0147 (13)
C5	0.045 (2)	0.045 (2)	0.054 (2)	−0.0166 (16)	0.0011 (16)	−0.0251 (17)
C6	0.054 (2)	0.0294 (17)	0.067 (3)	−0.0139 (16)	0.0035 (19)	−0.0152 (16)
C7	0.0356 (17)	0.0284 (16)	0.053 (2)	−0.0085 (13)	−0.0034 (15)	−0.0005 (14)
C8	0.057 (2)	0.0317 (18)	0.066 (3)	−0.0117 (17)	−0.005 (2)	0.0130 (17)
C9	0.056 (2)	0.052 (2)	0.045 (2)	−0.0155 (19)	−0.0094 (18)	0.0235 (18)
C10	0.0361 (18)	0.050 (2)	0.0353 (17)	−0.0103 (15)	−0.0067 (14)	0.0071 (15)
C11	0.057 (2)	0.075 (3)	0.0253 (17)	−0.014 (2)	−0.0086 (16)	0.0037 (17)
C12	0.062 (3)	0.074 (3)	0.0280 (17)	−0.021 (2)	−0.0083 (16)	−0.0125 (17)
C13	0.0382 (18)	0.0484 (19)	0.0346 (17)	−0.0101 (15)	−0.0039 (13)	−0.0165 (14)
C14	0.085 (3)	0.057 (2)	0.052 (2)	−0.030 (2)	−0.009 (2)	−0.0245 (19)
C15	0.0275 (15)	0.0337 (15)	0.0304 (15)	−0.0087 (12)	−0.0043 (12)	0.0016 (12)
C16	0.0304 (15)	0.0274 (14)	0.0335 (15)	−0.0106 (12)	−0.0021 (12)	−0.0006 (11)

### Geometric parameters (Å, °)

**Table:**

Ni—O3	2.046 (2)	C3—H3C	0.9600
Ni—O5	2.066 (2)	C4—C5	1.405 (5)
Ni—N2	2.076 (2)	C5—C6	1.352 (6)
Ni—N1	2.087 (2)	C5—H5A	0.9300
Ni—O1	2.148 (3)	C6—C7	1.406 (5)
Ni—O2	2.150 (2)	C6—H6A	0.9300
Ni—C1	2.457 (4)	C7—C16	1.403 (4)
O1—C1	1.244 (5)	C7—C8	1.429 (5)
O2—C1	1.249 (5)	C8—C9	1.341 (6)
C1—H1A	0.9300	C8—H8A	0.9300
O3—C2	1.235 (4)	C9—C10	1.428 (5)
O4—C2	1.233 (4)	C9—H9A	0.9300
C2—H2A	0.9300	C10—C15	1.399 (4)
O5—H52	0.8426	C10—C11	1.408 (5)
O5—H51	0.8597	C11—C12	1.354 (6)
O6—H61	0.8524	C11—H11A	0.9300
O6—H62	0.8469	C12—C13	1.406 (5)
N1—C4	1.337 (4)	C12—H12A	0.9300
N1—C16	1.370 (4)	C13—C14	1.495 (5)
N2—C13	1.334 (4)	C14—H14A	0.9600
N2—C15	1.359 (4)	C14—H14B	0.9600
C3—C4	1.497 (5)	C14—H14C	0.9600
C3—H3A	0.9600	C15—C16	1.444 (4)
C3—H3B	0.9600		
			
O3—Ni—O5	175.85 (9)	C4—C3—H3C	109.5
O3—Ni—N2	88.19 (10)	H3A—C3—H3C	109.5
O5—Ni—N2	90.52 (10)	H3B—C3—H3C	109.5
O3—Ni—N1	97.19 (10)	N1—C4—C5	121.0 (3)
O5—Ni—N1	86.51 (10)	N1—C4—C3	119.0 (3)
N2—Ni—N1	81.48 (10)	C5—C4—C3	119.9 (3)
O3—Ni—O1	89.42 (11)	C6—C5—C4	121.0 (3)
O5—Ni—O1	87.32 (11)	C6—C5—H5A	119.5
N2—Ni—O1	110.07 (11)	C4—C5—H5A	119.5
N1—Ni—O1	166.96 (10)	C5—C6—C7	119.6 (3)
O3—Ni—O2	88.37 (11)	C5—C6—H6A	120.2
O5—Ni—O2	92.29 (11)	C7—C6—H6A	120.2
N2—Ni—O2	170.43 (10)	C16—C7—C6	117.0 (3)
N1—Ni—O2	107.82 (10)	C16—C7—C8	119.6 (3)
O1—Ni—O2	60.97 (11)	C6—C7—C8	123.4 (3)
O3—Ni—C1	88.83 (13)	C9—C8—C7	121.2 (3)
O5—Ni—C1	89.66 (13)	C9—C8—H8A	119.4
N2—Ni—C1	140.41 (13)	C7—C8—H8A	119.4
N1—Ni—C1	138.01 (12)	C8—C9—C10	120.9 (3)
O1—Ni—C1	30.42 (12)	C8—C9—H9A	119.6
O2—Ni—C1	30.54 (12)	C10—C9—H9A	119.6
C1—O1—Ni	88.6 (2)	C15—C10—C11	117.1 (3)
C1—O2—Ni	88.4 (2)	C15—C10—C9	119.7 (3)
O1—C1—O2	122.0 (3)	C11—C10—C9	123.2 (3)
O1—C1—Ni	60.93 (19)	C12—C11—C10	119.6 (3)
O2—C1—Ni	61.03 (18)	C12—C11—H11A	120.2
O1—C1—H1A	119.0	C10—C11—H11A	120.2
O2—C1—H1A	119.0	C11—C12—C13	120.5 (3)
Ni—C1—H1A	179.7	C11—C12—H12A	119.8
C2—O3—Ni	121.6 (2)	C13—C12—H12A	119.8
O4—C2—O3	127.6 (3)	N2—C13—C12	121.0 (3)
O4—C2—H2A	116.2	N2—C13—C14	118.2 (3)
O3—C2—H2A	116.2	C12—C13—C14	120.8 (3)
Ni—O5—H52	116.4	C13—C14—H14A	109.5
Ni—O5—H51	111.9	C13—C14—H14B	109.5
H52—O5—H51	104.9	H14A—C14—H14B	109.5
H61—O6—H62	132.8	C13—C14—H14C	109.5
C4—N1—C16	118.4 (3)	H14A—C14—H14C	109.5
C4—N1—Ni	130.5 (2)	H14B—C14—H14C	109.5
C16—N1—Ni	110.88 (19)	N2—C15—C10	122.9 (3)
C13—N2—C15	118.9 (3)	N2—C15—C16	117.6 (3)
C13—N2—Ni	129.2 (2)	C10—C15—C16	119.5 (3)
C15—N2—Ni	111.95 (19)	N1—C16—C7	123.0 (3)
C4—C3—H3A	109.5	N1—C16—C15	118.0 (3)
C4—C3—H3B	109.5	C7—C16—C15	119.0 (3)
H3A—C3—H3B	109.5		

### Hydrogen-bond geometry (Å, °)

**Table:**

*D*—H···*A*	*D*—H	H···*A*	*D*···*A*	*D*—H···*A*
O5—H51···O4^i^	0.86	1.85	2.703 (3)	173.3
O5—H52···O6^ii^	0.84	2.03	2.808 (4)	154.2
O6—H61···O2^iii^	0.85	1.98	2.830 (4)	179.3
O6—H62···O3^iv^	0.85	2.43	3.077 (4)	133.4

Symmetry codes: (i) *x*+1, *y*, *z*; (ii) −*x*+2, −*y*, −*z*+1; (iii) *x*, *y*, *z*−1; (iv) −*x*+1, −*y*, −*z*+1.
